# Pyridine-4-carboximidamidate chloride

**DOI:** 10.1107/S160053680903517X

**Published:** 2009-09-09

**Authors:** Ping Fan, Lei Wang, Huidong Zhang

**Affiliations:** a College of Chemistry, Liaoning University, Shenyang 110036, People’s Republic of China; b Liaoning Fixed Star Chemicals (Group) Co. Ltd, Dandong 118000, People’s Republic of China

## Abstract

In the title salt, C_6_H_8_N_3_^+^·Cl^−^, each pyridine­carbox­imid­amidate cation is linked to two symmetry-related cations through N—H⋯N hydrogen bonds, and to two chloride ions by N—H⋯Cl hydrogen bonds. The N—H⋯N hydrogen bonds involve the pyridine N atom and one NH_2_ group. In the crystal, N—H⋯N and N—H⋯Cl hydrogen bonds extend the structure into two-dimensional layers. Weak C—H⋯Cl inter­actions further connect these layers into a three-dimensional network.

## Related literature

For background, see: Chudinov *et al.* (2005 ▶); Kamei *et al.* (2005 ▶).
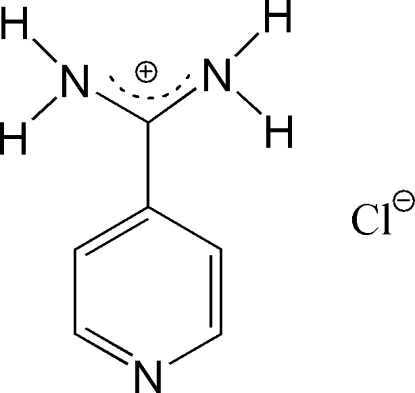


## Experimental

### 

#### Crystal data

C_6_H_8_N_3_^+^·Cl^−^
*M* *_r_* = 157.60
Orthorhombic, 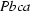

*a* = 7.3928 (13) Å
*b* = 10.4467 (16) Å
*c* = 18.925 (3) Å
*V* = 1461.6 (4) Å^3^
*Z* = 8
Mo *K*α radiation
μ = 0.44 mm^−1^
*T* = 293 K
0.37 × 0.32 × 0.21 mm

#### Data collection

Bruker SMART CCD area-detector diffractometer
Absorption correction: multi-scan (*SADABS*; Bruker, 2001 ▶) *T*_min_ = 0.853, *T*_max_ = 0.911
1949 measured reflections
1435 independent reflections
1215 reflections with *I* > 2σ(*I*)
*R*_int_ = 0.018

#### Refinement

*R*[*F* ^2^ > 2σ(*F* ^2^)] = 0.033
*wR*(*F* ^2^) = 0.092
*S* = 1.04
1435 reflections
124 parameters
All H-atom parameters refined
Δρ_max_ = 0.23 e Å^−3^
Δρ_min_ = −0.18 e Å^−3^

### 

Data collection: *SMART* (Bruker, 2001 ▶); cell refinement: *SAINT* (Bruker, 2001 ▶); data reduction: *SAINT*; program(s) used to solve structure: *SHELXS97* (Sheldrick, 2008 ▶); program(s) used to refine structure: *SHELXL97* (Sheldrick, 2008 ▶); molecular graphics: *SHELXTL* (Sheldrick, 2008 ▶); software used to prepare material for publication: *SHELXL97*.

## Supplementary Material

Crystal structure: contains datablocks I, global. DOI: 10.1107/S160053680903517X/bh2242sup1.cif

Structure factors: contains datablocks I. DOI: 10.1107/S160053680903517X/bh2242Isup2.hkl

Additional supplementary materials:  crystallographic information; 3D view; checkCIF report

## Figures and Tables

**Table 1:** Hydrogen-bond geometry (Å, °)

*D*—H⋯*A*	*D*—H	H⋯*A*	*D*⋯*A*	*D*—H⋯*A*
N2—H2*A*⋯N1^i^	0.88 (2)	2.22 (2)	3.058 (2)	160 (2)
N2—H2*B*⋯Cl1	0.83 (2)	2.79 (2)	3.476 (2)	142 (2)
N3—H3*A*⋯Cl1	0.93 (2)	2.19 (2)	3.100 (2)	167 (2)
N3—H3*B*⋯Cl1^ii^	0.89 (2)	2.41 (2)	3.270 (2)	161 (2)
C5—H5⋯Cl1^iii^	0.90 (2)	2.68 (2)	3.556 (2)	166 (2)

Symmetry codes: (i) 
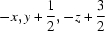
; (ii) 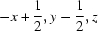; (iii) 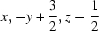.

**Fig. 1.**
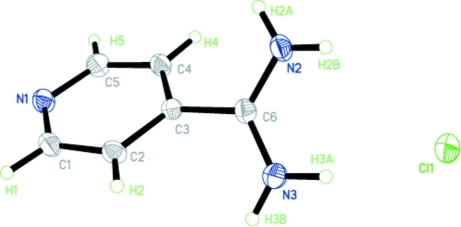
View of (I), showing atomic labels and displacement ellipsoids drawn at 30% probability level.

**Fig. 2.**
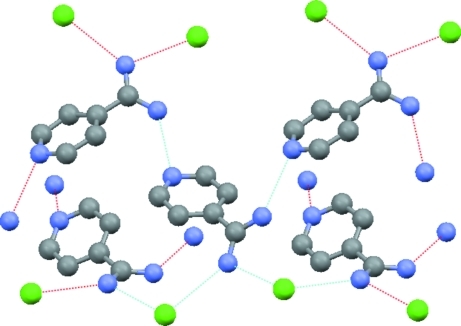
N—H···N and N—H···Cl hydrogen bonds in the crystal.

**Fig. 3.**
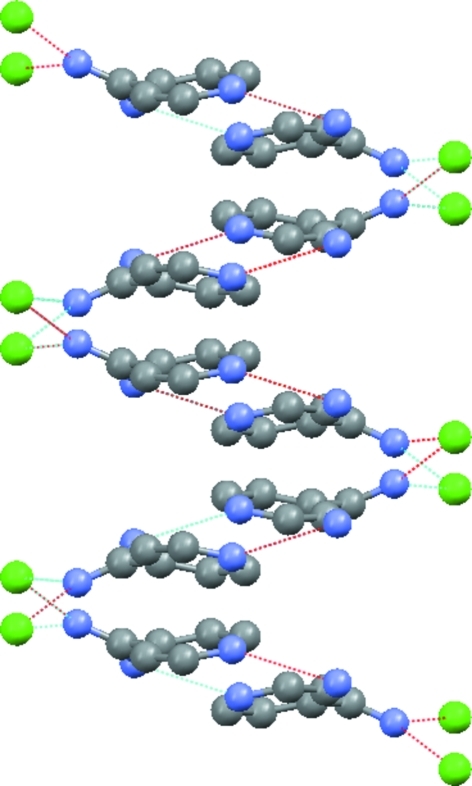
View of the hydrogen bonded one-dimensional chain along b axis.

## supplementary crystallographic information

### Comment

The title compound, also known as isonicotinamidine hydrochloride, served as a key intermediate in the synthesis of pharmacologically active compounds. It had attracted a great deal of interest during recent years. A series of new piperidinyl- and 1,2,3,6-tetrahydropyridinylpyrimidine derivatives was synthesized by using isonicotinamidine as an important intermediate. Isonicotinamidine has a unique structure and exists in the form of hydrochloride or acetate (Chudinov *et al.*, 2005; Kamei *et al.*, 2005).

The title compound is an organic salt (Fig. 1). In the cation, dihedral angle between the pyridyl ring and the plane confined by N2, N3 and C6 is 42.1°. Each isonicotinamidine cation is connected to two other cations by N—H···N hydrogen bonds, and to two Cl^-^ anions by N—H···Cl hydrogen bonds (Fig. 2), to form two dimensional layers including one-dimensional zigzag chains (Fig. 3). Weak C—H···Cl interactions [C···Cl = 3.556 (2) Å] link these layers to provide a three-dimensional supramolecular network.

### Experimental

The title compound was prepared according to the method of Kamei *et al.* (2005). Block-shaped crystals suitable for X-ray diffraction were obtained from ethanol/acetone.

### Refinement

H atoms were located from difference maps and freely refined.

### Crystal data

**Table:**

C_6_H_8_N_3_^+^·Cl^−^	*F*(000) = 656
*M**_r_* = 157.60	*D*_x_ = 1.432 Mg m^−^^3^
Orthorhombic, *P**b**c**a*	Mo *K*α radiation, λ = 0.71073 Å
Hall symbol: -P 2ac 2ab	Cell parameters from 542 reflections
*a* = 7.3928 (13) Å	θ = 2.3–22.8°
*b* = 10.4467 (16) Å	µ = 0.44 mm^−^^1^
*c* = 18.925 (3) Å	*T* = 293 K
*V* = 1461.6 (4) Å^3^	Block, colourless
*Z* = 8	0.37 × 0.32 × 0.21 mm

### Data collection

**Table:**

Bruker SMART CCD area-detector diffractometer	1435 independent reflections
Radiation source: fine-focus sealed tube	1215 reflections with *I* > 2σ(*I*)
graphite	*R*_int_ = 0.018
φ and ω scans	θ_max_ = 26.0°, θ_min_ = 2.2°
Absorption correction: multi-scan (*SADABS*; Bruker, 2001)	*h* = −9→1
*T*_min_ = 0.853, *T*_max_ = 0.911	*k* = −1→12
1949 measured reflections	*l* = −23→1

### Refinement

**Table:**

Refinement on *F*^2^	Secondary atom site location: difference Fourier map
Least-squares matrix: full	Hydrogen site location: inferred from neighbouring sites
*R*[*F*^2^ > 2σ(*F*^2^)] = 0.033	All H-atom parameters refined
*wR*(*F*^2^) = 0.092	*w* = 1/[σ^2^(*F*_o_^2^) + (0.0416*P*)^2^ + 0.5489*P*] where *P* = (*F*_o_^2^ + 2*F*_c_^2^)/3
*S* = 1.03	(Δ/σ)_max_ = 0.001
1435 reflections	Δρ_max_ = 0.23 e Å^−^^3^
124 parameters	Δρ_min_ = −0.17 e Å^−^^3^
0 restraints	Extinction correction: *SHELXL97* (Sheldrick, 2008), Fc^*^=kFc[1+0.001xFc^2^λ^3^/sin(2θ)]^-1/4^
Primary atom site location: structure-invariant direct methods	Extinction coefficient: 0.0056 (15)

### Fractional atomic coordinates and isotropic or equivalent isotropic displacement parameters (Å^2^)

**Table:**

	*x*	*y*	*z*	*U*_iso_*/*U*_eq_	
N1	0.0852 (2)	0.38353 (14)	0.75509 (8)	0.0367 (4)	
C1	0.0499 (3)	0.36114 (17)	0.82329 (10)	0.0367 (4)	
Cl1	0.15740 (8)	0.91636 (4)	1.05546 (2)	0.0432 (2)	
N2	0.0353 (2)	0.79417 (15)	0.89141 (9)	0.0369 (4)	
C2	0.0593 (3)	0.45387 (17)	0.87518 (9)	0.0341 (4)	
N3	0.1766 (3)	0.66264 (17)	0.97084 (9)	0.0428 (4)	
C3	0.1026 (2)	0.57807 (15)	0.85578 (9)	0.0285 (4)	
C4	0.1401 (3)	0.60305 (17)	0.78520 (9)	0.0337 (4)	
C5	0.1315 (3)	0.50297 (18)	0.73774 (9)	0.0376 (4)	
C6	0.1052 (2)	0.68338 (16)	0.90895 (9)	0.0310 (4)	
H4	0.171 (3)	0.6863 (18)	0.7679 (10)	0.033 (5)*	
H1	0.015 (3)	0.275 (2)	0.8348 (11)	0.044 (6)*	
H2	0.032 (3)	0.4327 (18)	0.9204 (11)	0.041 (5)*	
H5	0.159 (3)	0.519 (2)	0.6925 (12)	0.048 (6)*	
H2B	0.037 (3)	0.854 (2)	0.9202 (13)	0.060 (7)*	
H2A	−0.016 (3)	0.803 (2)	0.8500 (12)	0.050 (6)*	
H3B	0.229 (4)	0.590 (2)	0.9833 (12)	0.056 (7)*	
H3A	0.184 (3)	0.732 (2)	1.0012 (14)	0.061 (7)*	

### Atomic displacement parameters (Å^2^)

**Table:**

	*U*^11^	*U*^22^	*U*^33^	*U*^12^	*U*^13^	*U*^23^
N1	0.0452 (9)	0.0312 (8)	0.0338 (8)	0.0019 (7)	0.0001 (7)	−0.0051 (7)
C1	0.0439 (11)	0.0261 (9)	0.0400 (10)	−0.0002 (8)	−0.0007 (9)	0.0007 (7)
Cl1	0.0580 (3)	0.0357 (3)	0.0359 (3)	0.0007 (2)	−0.0055 (2)	−0.00566 (18)
N2	0.0484 (10)	0.0262 (8)	0.0360 (9)	0.0009 (7)	0.0006 (8)	−0.0043 (7)
C2	0.0436 (11)	0.0306 (9)	0.0280 (9)	0.0035 (8)	0.0027 (8)	0.0019 (7)
N3	0.0613 (12)	0.0348 (9)	0.0323 (8)	0.0070 (8)	−0.0090 (8)	−0.0067 (7)
C3	0.0313 (9)	0.0266 (8)	0.0277 (8)	0.0021 (7)	−0.0020 (7)	−0.0021 (7)
C4	0.0421 (10)	0.0280 (9)	0.0310 (9)	−0.0020 (8)	0.0003 (8)	0.0024 (7)
C5	0.0488 (12)	0.0377 (10)	0.0264 (9)	−0.0002 (8)	0.0022 (8)	−0.0012 (8)
C6	0.0348 (9)	0.0280 (9)	0.0300 (9)	−0.0018 (7)	0.0031 (7)	−0.0020 (7)

### Geometric parameters (Å, °)

**Table:**

N1—C5	1.335 (2)	N3—C6	1.303 (2)
N1—C1	1.337 (2)	N3—H3B	0.89 (2)
C1—C2	1.381 (3)	N3—H3A	0.93 (3)
C1—H1	0.96 (2)	C3—C4	1.389 (2)
N2—C6	1.310 (2)	C3—C6	1.491 (2)
N2—H2B	0.83 (3)	C4—C5	1.380 (3)
N2—H2A	0.88 (2)	C4—H4	0.958 (19)
C2—C3	1.386 (2)	C5—H5	0.90 (2)
C2—H2	0.91 (2)		
			
C5—N1—C1	116.80 (15)	C2—C3—C4	118.47 (16)
N1—C1—C2	123.63 (17)	C2—C3—C6	120.99 (15)
N1—C1—H1	115.7 (12)	C4—C3—C6	120.52 (15)
C2—C1—H1	120.6 (12)	C5—C4—C3	118.33 (17)
C6—N2—H2B	119.8 (17)	C5—C4—H4	118.5 (11)
C6—N2—H2A	119.2 (15)	C3—C4—H4	123.2 (11)
H2B—N2—H2A	121 (2)	N1—C5—C4	124.05 (17)
C1—C2—C3	118.68 (16)	N1—C5—H5	118.2 (15)
C1—C2—H2	119.3 (13)	C4—C5—H5	117.7 (15)
C3—C2—H2	122.0 (13)	N3—C6—N2	122.31 (17)
C6—N3—H3B	123.9 (15)	N3—C6—C3	119.28 (16)
C6—N3—H3A	116.9 (16)	N2—C6—C3	118.41 (16)
H3B—N3—H3A	119 (2)		

### Hydrogen-bond geometry (Å, °)

**Table:**

*D*—H···*A*	*D*—H	H···*A*	*D*···*A*	*D*—H···*A*
N2—H2A···N1^i^	0.88 (2)	2.22 (2)	3.058 (2)	160 (2)
N3—H3A···Cl1	0.93 (2)	2.19 (2)	3.100 (2)	167 (2)
N2—H2B···Cl1	0.83 (2)	2.79 (2)	3.476 (2)	142 (2)
N3—H3B···Cl1^ii^	0.89 (2)	2.41 (2)	3.270 (2)	161 (2)
C5—H5···Cl1^iii^	0.90 (2)	2.68 (2)	3.556 (2)	166 (2)

Symmetry codes: (i) −*x*, *y*+1/2, −*z*+3/2; (ii) −*x*+1/2, *y*−1/2, *z*; (iii) *x*, −*y*+3/2, *z*−1/2.
